# Summary of the Transactions of the College of Physicians of Philadelphia

**Published:** 1851-07

**Authors:** 


*Summary of the Transactions of the College of Physicians of Philadelphia.* — This is a valuable quarterly publication, the circulation of which has hitherto been quite limited. By a new arrangement, the work will be issued by Messrs. Lippincott, Grambo & Co., in an improved style of typography, at one dollar *per annum.* Each number will contain from 60 to 100 pages. The extent to which we have been accustomed to draw upon the work in selecting matter for the Eclectic department of this Journal, is the best evidence we can furnish of the value we set upon it.

